# CT perfusion during delayed cerebral ischemia after subarachnoid hemorrhage: distinction between reversible ischemia and ischemia progressing to infarction

**DOI:** 10.1007/s00234-015-1543-3

**Published:** 2015-06-02

**Authors:** Charlotte H. P. Cremers, Pieter C. Vos, Irene C. van der Schaaf, Birgitta K. Velthuis, Mervyn D. I. Vergouwen, Gabriel J. E. Rinkel, Jan Willem Dankbaar

**Affiliations:** Department of Neurology and Neurosurgery, Brain Center Rudolf Magnus, University Medical Center Utrecht, PO Box 85500, 3508 GA Utrecht, Utrecht The Netherlands; Department of Radiology, University Medical Center Utrecht, Utrecht, The Netherlands; Image Sciences Institute, University Medical Center Utrecht, Utrecht, The Netherlands

**Keywords:** CT perfusion, Delayed cerebral ischemia, Subarachnoid hemorrhage

## Abstract

**Introduction:**

Delayed cerebral ischemia (DCI) after aneurysmal subarachnoid hemorrhage (aSAH) can be reversible or progress to cerebral infarction. In patients with a deterioration clinically diagnosed as DCI, we investigated whether CT perfusion (CTP) can distinguish between reversible ischemia and ischemia progressing to cerebral infarction.

**Methods:**

From a prospectively collected series of aSAH patients, we included those with DCI, CTP on the day of clinical deterioration, and follow-up imaging. In qualitative CTP analyses (visual assessment), we calculated positive and negative predictive value (PPV and NPV) with 95 % confidence intervals (95%CI) of a perfusion deficit for infarction on follow-up imaging. In quantitative analyses, we compared perfusion values of the least perfused brain tissue between patients with and without infarction by using receiver-operator characteristic curves and calculated a threshold value with PPV and NPV for the perfusion parameter with the highest area under the curve.

**Results:**

In qualitative analyses of 33 included patients, 15 of 17 patients (88 %) with and 6 of 16 patients (38 %) without infarction on follow-up imaging had a perfusion deficit during clinical deterioration (*p* = 0.002). Presence of a perfusion deficit had a PPV of 71 % (95%CI: 48–89 %) and NPV of 83 % (95%CI: 52–98 %) for infarction on follow-up. Quantitative analyses showed that an absolute minimal cerebral blood flow (CBF) threshold of 17.7 mL/100 g/min had a PPV of 63 % (95%CI: 41–81 %) and a NPV of 78 % (95%CI: 40–97 %) for infarction.

**Conclusions:**

CTP may differ between patients with DCI who develop infarction and those who do not. For this purpose, qualitative evaluation may perform marginally better than quantitative evaluation.

## Introduction

Delayed cerebral ischemia (DCI) is a severe complication of aneurysmal subarachnoid hemorrhage (aSAH). DCI affects approximately 20–30 % of patients who survive the initial hemorrhage and usually occurs between 4 and 14 days after the hemorrhage [1, 2]. Clinical deterioration from DCI can present as a new focal neurologic impairment, a decrease in the level of consciousness, or both [3]. DCI can be reversible or progress to cerebral infarction, which increases the risk of poor functional outcome [4–6]. DCI is mainly a clinical diagnosis at onset based on exclusion of other causes of the deterioration and confirmed by follow-up imaging showing cerebral infarction, but recent studies have shown that CT perfusion (CTP) can be helpful to detect DCI at the time of clinical deterioration [7]. However, it remains unknown if patients with reversible ischemia can be distinguished from patients with ischemia progressing to cerebral infarction. Such differentiation would be useful for clinical trials testing the efficacy of interventions to prevent cerebral infarction. DCI can be assessed with CTP in different ways: qualitatively (with visual detection of a perfusion deficit) and with quantitative thresholds (both absolute and relative).

The purpose of this study was to investigate whether CTP can distinguish between reversible ischemia and ischemia progressing to cerebral infarction.

## Methods

### Design

This study was approved by the institutional review board. Patients were selected from a prospectively collected series of consecutive aSAH patients admitted to our hospital between August 2007 and November 2010. In our hospital, all patients with aSAH routinely undergo multimodal CT, consisting of non-contrast CT (NCCT), CTP, and CT-angiography (CTA), on admission and at the time of clinical deterioration after aSAH. Inclusion criteria for this study were (1) 18 years of age or older, (2) clinical deterioration due to DCI within 21 days after aSAH, (3) NCCT and CTP imaging at the onset of clinical deterioration, and (4) follow-up imaging (CT or MRI) at least 3 days after onset of clinical deterioration. Exclusion criteria were pregnancy, impaired renal function (creatinine >200 μmol/L) or other contraindications for contrast administration, and CTP scans with extensive movement artifacts. In our institution, none of the SAH patients are treated with endovascular rescue therapy.

### DCI

Clinical deterioration due to DCI was defined as a clinical deterioration (new focal deficit or decrease in Glasgow Coma Scale of at least two points on the total score or one of its individual components or both) lasting 1 h or longer with no evidence for rebleeding or hydrocephalus on CT and no other medical causes, such as cardiovascular or pulmonary complications, infections, or metabolic disturbances [3].

Cerebral infarction due to DCI was defined as a new infarction on follow-up CT or MRI that was not present on earlier imaging and could not be attributed to other causes such as aneurysm treatment, ventricular catheter placement, or intraparenchymal hematoma [3].

### CTP imaging

All imaging studies were performed on a 16-, 64-, or 128-multidetector CT scanner (Philips Mx8000 IDT 16, Philips Brilliance 16P, Philips Brilliance 64, Philips Brilliance iCT; Best, the Netherlands). For the CTP scan, 40 ml of non-ionic contrast agent (lopromide, Ultravist, 300 mg iodine/ml, Schering, Berlin, Germany) was injected into the cubital vein (18-gauge needle) at a rate of 5 ml/s followed by a 40 ml saline flush at a rate of 5 ml/s using a dual power injector (Stellant Dual CT injector, Medrad Europe BV, Beek, the Netherlands). The following parameters were used: 16 slice, 90 kVp, 150 mAs, 8 × 3 mm collimation; 64 slice, 80 kVp, 150 mAs, 64 × 0.625 mm collimation; 128 slice, 80 kVp, 150 mAs, 128 × 0.625 mm collimation. All scanners used a 512 × 512 matrix, a field of view ranging from 160 to 220 mm, UB filter and standard resolution and acquired one image per 2 s during 60 s.

### CTP post-processing

CT perfusion maps were generated using an open source software package (Perfusion Mismatch Analyzer, PMA version 4.0.4.4, ASIST Japan). The optimal arterial input function (AIF) and venous output function (VOF) were automatically selected by the software and corrected manually if necessary. Patient movement was corrected with Elastix, and for noise reduction, a filter using the time-intensity profile similarity to reduce noise in the spatial domain was applied [8, 9]. Quantitative cerebral perfusion values were calculated using block-circulant singular value decomposition (bSVD), which is described as a tracer delay-insensitive algorithm [10]. CTP maps were created for cerebral blood flow (CBF), cerebral blood volume (CBV), mean transit time (MTT), and time to peak (TTP).

An in-house software tool was developed using Mevislab (®MevisLab, software for medical image processing and visualization; http://www.mevislab.de) for visualization of the perfusion maps and to extract statistics from regions of interest (ROIs) that were manually placed on the perfusion maps.

### CTP evaluation

CT perfusion maps were evaluated qualitatively (visual assessment) and quantitatively. Qualitative evaluation was done by one observer (JWD, 8-year experience with CTP in DCI) who was blinded for follow-up imaging and the final diagnosis but with knowledge of the patient’s clinical condition at the time of imaging (GCS-scores and focal deficit if applicable). For every patient, the four CTP maps were displayed together with the NCCT. Positive findings were hypoperfused areas (lower CBF or CBV or higher MTT or TTP), which were not localized in the neurosurgical trajectory, directly surrounding an intracerebral hematoma, or caused by an endovascular intervention.

**Fig. 1:**
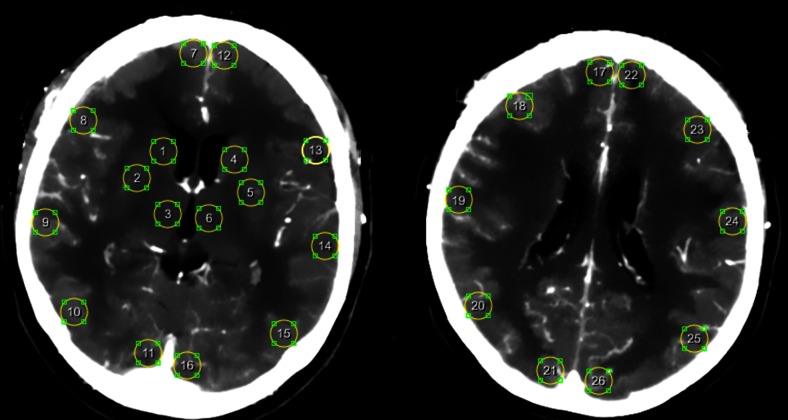
Standard ROIs in the cortical flow territories of the anterior cerebral artery, middle cerebral artery, and posterior cerebral artery and in the basal ganglia

For quantitative analyses, we investigated the least perfused region of the brain by selecting the lowest CBF and CBV value and the highest MTT and TTP value. The least perfused region was selected from standard ROIs and perfusion deficits (if applicable):

1. Standard ROIs were drawn in the cortical flow territories of the anterior cerebral artery (ACA), middle cerebral artery (MCA), and posterior cerebral artery (PCA) and in the basal ganglia (Fig. 1). Because perfusion in unaffected white matter can be lower than perfusion in a perfusion deficit in gray matter, we did not include white matter regions in the standard ROIs.
2. Perfusion deficits. In all patients with a visible perfusion deficit on CTP during clinical deterioration, the deficit was manually delineated. Delineation was done blinded for the results of follow-up imaging.

For relative perfusion values, the least perfused ROI was compared to the contralateral ROI. Subsequently, for CBF and CBV minimal ratios of cerebral perfusion values were calculated and for MTT and TTP maximal differences of perfusion values were calculated.

### Analyses

For the qualitative analyses, we calculated positive and negative predictive values (PPV and NPV) with corresponding 95 % confidence intervals (95 % CI) of presence of perfusion deficits for infarction on follow-up. Subsequently, we determined the proportion of patients with a perfusion deficit on CTP during clinical deterioration for patients with and without an infarction on follow-up imaging. Differences between groups were analyzed using the chi-square test.

For the quantitative analyses, we constructed a receiver-operator characteristic (ROC) curve for the distinction between patients with and without an infarction on follow-up imaging for all parameters and calculated the area under the curve (AUC). Of all perfusion parameters, the parameter with the highest AUC was selected and, subsequently, PPV and NPV (with 95 % CI) for the threshold value with the optimal diagnostic cutoff point were calculated.

## Results

We included 33 patients with clinical deterioration due to DCI, of which 17 patients had an infarction on follow-up imaging (Table 1). Four patients were scanned on a 16-multidetector CT scanner, 18 patients on a 64-multidetector CT scanner, and 11 patients on a 128-multidetector CT scanner.

**Table 1:** Patient characteristics

	DCI with infarction (*n* = 17)	DCI without infarction (*n* = 16)
Women (n, %)	12 (71 %)	14 (88 %)
Age (mean, range)	52 (29–67)	58 (28–81)
Admission WFNS score (%)		
I	7 (41 %)	4 (25 %)
II	5 (29 %)	5 (31 %)
III	2 (12 %)	1 (6 %)
IV	1 (6 %)	4 (25 %)
V	2 (12 %)	2 (13 %)
Aneurysm location (%)		
Acom, ACA	8 (47 %)	7 (44 %)
Pcom	3 (18 %)	5 (31 %)
BA	3 (18 %)	2 (13 %)
ICA	3 (18 %)	2 (13 %)
Treatment (%)		
Coil	10 (59 %)	9 (56 %)
Clip	6 (35 %)	6 (38 %)
None	0 (0 %)	1 (6 %)
Both	1 (6 %)	0 (0 %)
Hypertension induction as treatment for DCI (%)	5 (29 %)	4 (25 %)
Perfusion deficit visible on CTP during clinical deterioration (%)	15 (88 %)	6 (38 %)
Clinical presentation at time of clinical deterioration (%)		
WFNS score		
I	0 (0 %)	0 (0 %)
II	1 (6 %)	1 (6 %)
III	1 (6 %)	1 (6 %)
IV	6 (35 %)	3 (19 %)
V	2 (12 %)	5 (31 %)
Focal deficit	6 (35 %)	5 (31 %)
Unknown	1 (6 %)	1 (6 %)
Mean CTP value (with SD) per patient (standard ROIs and perfusion deficits)		
CBF (mL/100 g/min)	25.29 (9.76)	30.38 (12.43)
CBV (mL/100 g)	2.79 (0.98)	3.21 (1.15)
MTT (s)	14.43 (1.97)	13.88 (1.07)
TTP (s)	14.20 (3.04)	14.09 (2.09)

*DCI* delayed cerebral ischemia, *WFNS* World Federation of Neurosurgeons, *Acom* anterior communicating artery, *ACA* anterior cerebral artery, *Pcom* posterior communicating artery, *BA* basilar artery, *ICA* internal carotid artery, *CTP* CT perfusion, *SD* standard deviation, *ROIs* regions of interest, *CBF* cerebral blood flow, *CBV* cerebral blood volume, *MTT* mean transit time, *TTP* time to peak

### Qualitative analyses

A perfusion deficit was seen on CTP during clinical deterioration in 15 of 17 patients (88 %) with a final infarction on follow-up imaging compared to 6 of 16 patients (38 %) without a final infarction (*p* = 0.002) (Table 1). The PPV for the development of an infarction was 71 % (95%CI: 48–89 %) and the NPV was 83 % (95%CI: 52–98 %).

### Quantitative analyses

For the least perfused region of the brain, median perfusion values (with interquartile ranges (IQR)) for DCI patients with and without an infarction on follow-up are presented in Table 2. A trend towards lower CBF and CBV and longer MTT and TTP was seen in patients with infarction compared to patients without an infarction on follow-up imaging. The AUC of the ROC curve for absolute CBF was 0.65, for CBV 0.58, for MTT 0.60, and for TTP 0.50. For the relative perfusion values, the AUCs were for CBF 0.52, CBV 0.47, MTT 0.57, and for TTP 0.52. Since the absolute CBF had the highest AUC, this parameter was selected for further analysis. An absolute CBF threshold of 17.7 mL/100 g/min showed a PPV of 63 % (95%CI: 41–81 %) and a NPV of 78 % (95%CI: 40–97 %).

**Table 2:** Lowest perfusion values

CTP parameter	Infarction at follow-up (*n* = 17)	No infarction at follow-up (*n* = 16)
Absolute values (median (IQR))		
CBF (mL/100 g/min)	11.66 (8.34–16.32)	16.08 (8.92–23.57)
CBV (mL/100 g)	1.66 (1.07–2.14)	1.78 (1.23–2.44)
MTT (s)	21.72 (16.76–26.54)	18.83 (15.65–23.05)
TTP (s)	19.18 (14.86–23.76)	18.24 (16.29–21.94)
Relative values (median (IQR))		
CBF ratio	0.53 (0.42–0.60)	0.56 (0.36–0.62)
CBV ratio	0.57 (0.53–0.65)	0.59 (0.45–0.65)
MTT difference (s)	7.62 (2.96–12.10)	4.68 (2.29–9.44)
TTP difference (s)	3.77 (2.16–8.92)	4.46 (1.77–8.01)

Lowest perfusion values: lowest value and ratio in CBF and CBV and highest value and difference in MTT and TTP, measured in standard ROIs in gray matter and basal ganglia or perfusion deficits (if applicable)

*CTP* CT perfusion, *n* number, *IQR* interquartile range (25–75 %), *CBF* cerebral blood flow, *CBV* cerebral blood volume, *MTT* mean transit time, *TTP* time to peak.

## Discussion

The results of our study show that in patients with clinical deterioration due to DCI, qualitative CTP assessment predicts better an infarction on follow-up than quantitative assessment; however, the predictive values of perfusion deficits are only moderate. Patients with a visually assessed perfusion deficit at the time of clinical deterioration more often have an infarction on follow-up imaging than such patients without a perfusion deficit.

The distinction between reversible ischemia and ischemia progressing to infarction in patients with clinical deterioration due to DCI has been studied only once before in a subgroup analysis of a study investigating the value of CTP in predicting outcome after aSAH [11]. In that study, perfusion scans were visually assessed and additionally quantitative measurements were performed in standard ROIs. In contrast to our results, that study found no significant difference in the qualitative analyses of patients with and without an infarction, although a trend was seen to a higher proportion of visually assessed CTP deficits in DCI patients with infarction on follow-up compared to DCI patients without infarction [11]. In that study, 15 patients with infarction and 33 patients without infarction were included. Different software packages were used in both studies to generate the perfusion maps, which might explain the different results that were found. Therefore, standardized methods for measuring perfusion with CTP after SAH are needed [7]. In the quantitative analyses of that study also no significant differences were found between DCI patients with and without an infarction. Our study design for the quantitative analyses differs essentially from the previous study. We measured perfusion in the area with the lowest perfusion in each patient in a visually delineated perfusion deficit or in the absence of a perfusion deficit in the lowest standard ROI. In contrast, the previous study measured perfusion either in standard ROIs located within a perfusion deficit or used, in those patients without a perfusion deficit, the mean value of all standard ROIs instead of the ROI with lowest perfusion [11]. In our opinion, the comparison of the measurements in a perfusion deficit to the lowest values in patients without deficits is more realistic if quantitative differences between the two groups are being evaluated.

The absolute perfusion values found in our study are lower compared to perfusion values found in other studies in DCI patients [12, 13]. This can be explained by the fact that we measured perfusion in perfusion deficits and used the least perfused region, whereas in other studies the mean value of standard ROIs was used. In addition, quantitative perfusion values may differ due to differences in CTP algorithms [14]. Because absolute perfusion values are known to vary and may be influenced by user-dependent post-processing steps [14, 15], we also evaluated relative CTP measurements and found comparable results to the absolute CTP measurements.

In patients with ischemic stroke, a prediction map based on quantitative thresholds is used to distinguish between reversible and irreversible ischemic tissue [16]. Since we found lower predictive values for the quantitative thresholds compared to the qualitative assessment in this study, the development of a prediction map in DCI patients seems less useful than visual assessment.

Although the PPV and NPV of the visual assessment with CTP found in this study were not overwhelming, the visual assessment of CTP could be useful in clinical practice to give an indication of the chance of developing an infarction due to DCI. However, the predictive values of the visual assessment of CTP to distinguish between DCI patients with and without infarction need to be validated in a prospective study. In addition, its value for predicting outcome needs to be evaluated.

Other techniques like diffusion-weighted MRI may be more suited to detect irreversible ischemia in an early stage [17]. However, the use of MRI is often challenging in SAH patients because of incompatibility of monitoring equipment with the MR suite. In addition, in many hospitals, MRI is still not available around the clock. Several technical improvements for CTP imaging are currently being investigated [18–20]. This may make more accurate estimation of irreversible ischemia possible in the future.

Some limitations need to be addressed. Although the definition for DCI used in our study is widely accepted [3], the diagnosis of DCI is based on excluding other causes of clinical deterioration. Since there are many factors influencing the occurrence of clinical deterioration after aSAH, there may still be some misclassification, which may influence predictive values. Another potential limitation is that the qualitative assessment of the CTP scans was done by a single observer. In a previous study, lower test characteristics were found for a less experienced observer compared to an experienced observer; however this was based on only two observers [21]. In stroke studies, good reproducibility was found for visual assessment of CTP images [22]. Finally, although the percentage of patients receiving induced hypertension was similar in patients with and without an infarct on follow-up, the change in blood pressure and thereby cerebral blood flow may not have been equal. This may have introduced some bias. On the other hand, there are no data available in the literature on the effect of induced hypertension on the development of infarction.

To conclude, CTP may differ between patients with DCI who develop infarction and those who do not, but before these findings can be implemented in clinical practice, they should be confirmed in different patient populations in different centers. To differentiate between the two groups, qualitative analysis may perform marginally better than quantitative analysis.
